# A nutrient-responsive cancer-on-chip model for assessing chemotherapy responses under glucose restriction

**DOI:** 10.3389/fbioe.2026.1857650

**Published:** 2026-07-02

**Authors:** Andrea Giannoccaro, Maria Elisabetta Federica Palamà, Gianluca Ciardelli, Maurizio Aiello, Silvia Scaglione

**Affiliations:** 1 Politecnico di Torino, Dipartimento di Ingegneria Meccanica e Aerospaziale, Turin, Italy; 2 React4Life S.p.A., Genoa, Italy; 3 Istituto di Elettronica e di Ingegneria dell’Informazione e delle Telecomunicazioni Consiglio Nazionale delle Ricerche Sede di Genova, Genoa, Italy

**Keywords:** 3D tumor model, breast cancer, cancer-on-chip, fasting-mimicking diet, microphysiological system (MPS)

## Abstract

Fasting-mimicking diets have emerged as promising adjuvant strategies to enhance chemotherapy efficacy. However, conventional preclinical models do not adequately reproduce the microenvironmental and transport-related conditions associated with nutrient modulation, limiting mechanistic investigation of fasting-related therapeutic responses. Here, we developed a nutrient-responsive cancer-on-chip (CoC) platform integrating 3D human breast cancer models with systemic-like cisplatin administration under defined glucose conditions (high, low, no glucose). Complete glucose deprivation markedly reduced metabolic activity and cell viability, while mild reduction was largely tolerated, reflecting tumor metabolic adaptability. Tumor models were exposed to glucose modulation and cisplatin, with or without 24 h glucose-free pre-conditioning, in static and dynamic 3D systems. Static cultures displayed limited treatment response, whereas CoC exhibited significantly increased cell death, highlighting the limited predictive capacity of conventional models. Glucose-free pre-conditioning enhanced chemotherapy sensitivity, while fasting initiated concurrently with treatment produced minimal impact. Glucose reintroduction after glucose-free pre-conditioning impaired metabolic recovery, revealing a transient vulnerability window. Importantly, in healthy fibroblasts, glucose deprivation reduced metabolic activity without markedly affecting viability. A qualitative comparison with published clinical data from fasting-mimicking diet trials indicated that dynamic CoC models reproduced several treatment-related response trends more consistently than static systems. These findings suggest the potential of nutrient-responsive CoC systems as complementary tools for investigating context-dependent responses to fasting-associated therapeutic strategies.

## 1 Introduction

Triple-negative breast cancer (TNBC) represents a clinically challenging and biologically complex subtype of breast cancer, characterized by the lack of common hormone and growth factor receptors typically targeted in standard therapies (Yin et al., 2020). Although TNBC accounts for only 10%–20% of breast cancer diagnoses, it is disproportionately associated with early recurrence and poor clinical outcomes. Its molecular profile renders TNBC unresponsive to endocrine or HER2-targeted therapies, leaving cytotoxic chemotherapy as the primary treatment option (De Laurentiis et al., 2010). However, its aggressive nature and frequent development of resistance to therapies, including platinum-based agents like cisplatin, underscore the urgent need for novel therapeutic strategies (Zagami and Carey, 2022; Galluzzi et al., 2012).

Most cancerous cells, including TNBC, selectively depend on glucose metabolism for their survival (the Warburg effect) (Nencioni et al., 2018). This observation has catalyzed interest in dietary strategies, particularly glucose restriction and fasting-mimicking protocols, as potential adjuvants to conventional chemotherapy (Lv et al., 2014). Several studies suggest that such interventions may induce cellular stress and reduce cancer cells proliferation and motility, potentially enhancing the efficacy of cytotoxic drugs (Khajah et al., 2022; Kang et al., 2023), while mitigating its adverse effects on healthy tissues (Menyhárt and Győrffy, 2024; Kanarek et al., 2020; Qian et al., 2022). However, fasting-mimicking diets induce complex systemic endocrine, metabolic, and immune alterations that cannot be fully reproduced in simplified *in vitro* systems. In this context, reductionist *in vitro* approaches may be particularly useful to isolate and investigate specific microenvironmental components, such as extracellular nutrient availability. Nevertheless, reliable and reproducible *in vitro* validation of combined nutrient and chemotherapeutic effects remains limited. Traditional 2D cell culture systems fail to capture key aspects of tissue architecture, nutrient gradients, and drug transport, whereas animal models present intrinsic limitations related to species-specific metabolism, systemic physiology, and tumor evolution (Pound and Ritskes-Hoitinga, 2018; Mak et al., 2014).

To address this gap, three-dimensional (3D) fully humanized cell cultures have emerged as more biologically relevant models, offering an improved approximation of the complexity of tumor architecture compared to 2D systems (Hickman et al., 2014). Moreover, their integration with organ-on-chip (OoC) technologies further enhances physiological accuracy by introducing dynamic perfusion and related induced shear stress, and controlled drug delivery. Collectively, these features enable a closer approximation of *in vivo* tumor conditions, thereby supporting the predictive validity of preclinical assessments in a controlled setting (Xu et al., 2014; Wikswo, 2014).

Here, we present an *in vitro* TNBC model incorporating 3D alginate-based hydrogels cultured under both static and fluid-dynamic conditions taking advantage of the patented MIVO® cancer-on-chip platform (Marrella et al., 2021; Marzagalli et al., 2022; Fallarini et al., 2025). This platform has been already successfully validated in comparison with animal model to assess and quantify the ovarian tumor regression after chemotherapy administration, reproducing an intravenous-like drug delivery route.

Beyond mere delivery, the transition from static to controlled fluid flow dynamic conditions fundamentally alters the effective concentration of solutes reaching the cells: while static systems rely solely on passive diffusion, often hindered by the accumulation of metabolic by-products, cellular debris and extracellular presence, dynamic perfusion ensures a constant supply of nutrients and drugs, maintaining a stable concentration gradient across the clinically relevant-sized 3D tumor constructs and overcoming the transport limitations typical of static hydrogel cultures (Marrella et al., 2021).

In this wok, the effects of varying glucose concentrations on cancer cells viability and metabolism were evaluated to identify culture conditions capable of reproducing defined glucose-restriction states associated with fasting-like interventions. Subsequently, the combined effects of glucose restriction and cisplatin administration were investigated to explore how nutrient availability modulates chemotherapy response in a dynamic 3D TNBC model.

## 2 Results

### 2.1 Glucose deprivation but not glucose reduction impaired cancer cell metabolism in 2D *in vitro* TNBC model

Given the strong reliance of cancer cells on glucose metabolism, this study investigated the effects of glucose availability, from mild to severe deprivation, as an extreme metabolic stress condition, on cell viability and metabolic activity in *vitro* TNBC models (Khajah et al., 2022; Kang et al., 2023). ^7,8^MDA-MB-231 cells were cultured in High glucose (High G), Low glucose (Low G) or No glucose (No G) media, to model control, glucose reduction and glucose deprivation conditions, respectively.

**FIGURE 1:**
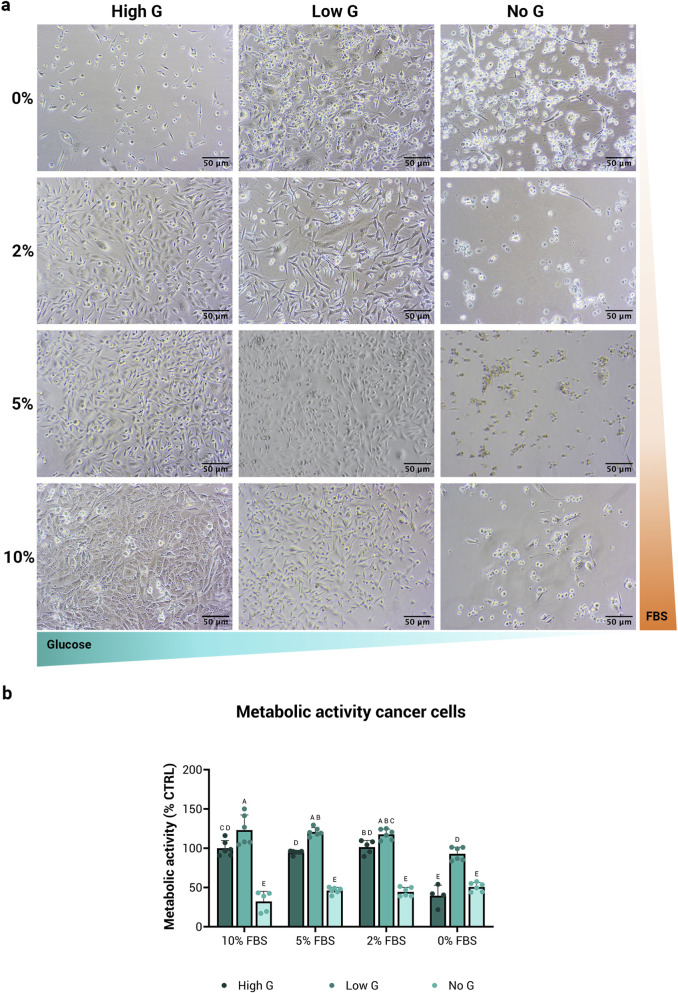
Effects of different FBS concentrations in fasting conditions. **(a)** Representative images of MDA-MB-231 cells cultured in DMEM High glucose (High G), DMEM Low glucose (Low G) or DMEM No glucose (No G), supplemented with 0%, 2%, 5% or 10% FBS. Scale bar: 50 μm. **(b)** Metabolic activity assessed by AlamarBlue. Data are reported as mean ± S.D. and expressed as percentage of control (High G, 10% FBS). Values with shared letters are not significantly different (p > 0.05) according with two-way ANOVA. N = 3.

To assess whether serum levels modulate glucose-dependent biological responses, cells were cultured in monolayer (2D) in High G, Low G or No G media, supplemented with 0%, 2%, 5% or 10% FBS. After 3 days, comparable morphology was observed among 2%, 5% and 10% groups under same glucose conditions (Figure 1a). In addition, High G and Low G cultures exhibited higher cell abundance and elongation, whereas No G groups displayed visibly fewer, rounded and poorly adherent cells. As expected, all 0% FBS conditions showed poor viability, regardless of glucose level.

These observations were consistent with AlamarBlue measurements (Figure 1b). Results showed that glucose reduction (Low G) did not affect cell metabolism, while glucose deprivation (No G) determined a >50% reduction compared to control condition (High G), showing similar trend across different FBS concentrations. In contrast, 0% FBS severely impaired metabolic activity even under High G and Low G conditions, masking glucose-specific effects.

Overall, results indicate that glucose availability markedly influences cellular morphology, viability and metabolism, with serum availability acting as a key modulator of the cellular response. Based on these observations, 10% FBS was selected for subsequent experiments to ensure stable baseline conditions and minimize confounding effects associated with serum deprivation. Importantly, serum supplementation was maintained during glucose modulation to avoid complete nutrient starvation and isolate, as much as possible, the contribution of extracellular glucose availability.

### 2.2 Glucose deprivation reduced cell viability and metabolic activity in 3D cancer models

**FIGURE 2:**
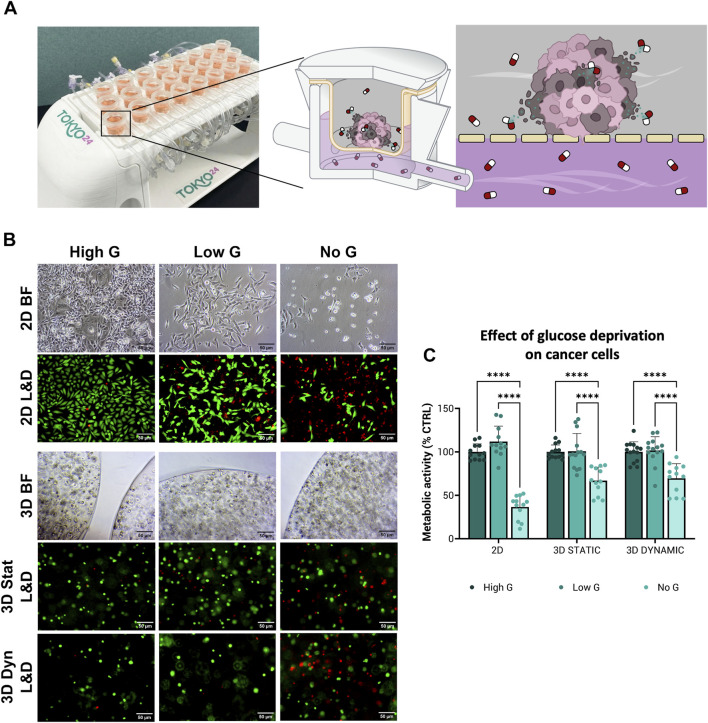
Effects of glucose deprivation on cancer cell viability and metabolic activity. **(A)** Schematic representation of MIVO(R) cancer-on-chip platform. **(B)** Representative images in BF and Live/Dead staining of MDA-MB-231 cells in 2D and 3D conditions cultured in High glucose (High G), Low glucose (Low G) or No glucose (No G) containing media for 3 days. Scale bar: 50 μm. **(C)** Metabolic activity assessed by AlamarBlue assay. Semi-quantitative data are reported as mean ± SD and expressed as percentage of control (High G). Statistical comparison was made using two-way ANOVA (N = 4), ****p < 0.0001.

To study the effects of glucose restriction in a more structured 3D environment compared to conventional 2D cultures, MDA-MB-231 cells were embedded in alginate-based hydrogels, while 2D monolayers were used as controls. Three-dimensional models were maintained under static or dynamic culture conditions. Specifically, the previously developed MIVO® Single Flow millifluidic device was adopted for the culture of 3D models under physiological dynamic flow (Figure 2A).

All models were cultured in High G, Low G or No G media, and after 3 days, cell viability and metabolic activity (normalized to untreated controls, serving as a relative indicator of cellular viability and proliferation) were assessed *via* Live/Dead assay (Figure 2B) and Alamar Blue assay (Figure 2C).

In 2D cultures, decreasing glucose levels induced a progressive reduction in viable (green-stained) cells. No G condition showed the most pronounced impairment, with few adherent cells and a predominance of rounded, detached, and dead (red-stained) cells. In contrast, High G cultures displayed abundant viable cells with minimal cells death.

In 3D alginate models, cells appeared with the expected rounded morphology. However, Live/Dead analysis revealed increased cell death under glucose deprivation, particularly in the No G dynamic 3D model.

Quantification of metabolic activity (Figure 2C) was consistent with these observations: in 2D cultures, No G caused an approximately 50% reduction compared to High G (p < 0.0001), while Low G showed minimal impact on cell metabolism. A similar trend was observed in both static and dynamic 3D models, although 3D cultures retained slightly higher metabolic function under No G, suggesting a greater resistance to nutrient deprivation.

These results indicate that glucose deprivation significantly impairs cell viability and viability-associated metabolic readouts in both 2D and 3D systems, while 3D models displayed enhanced resilience to nutrient scarcity compared to conventional 2D cultures.

### 2.3 Glucose deprivation induced stress-related pathways in both 2D and 3D cancer models

**FIGURE 3:**
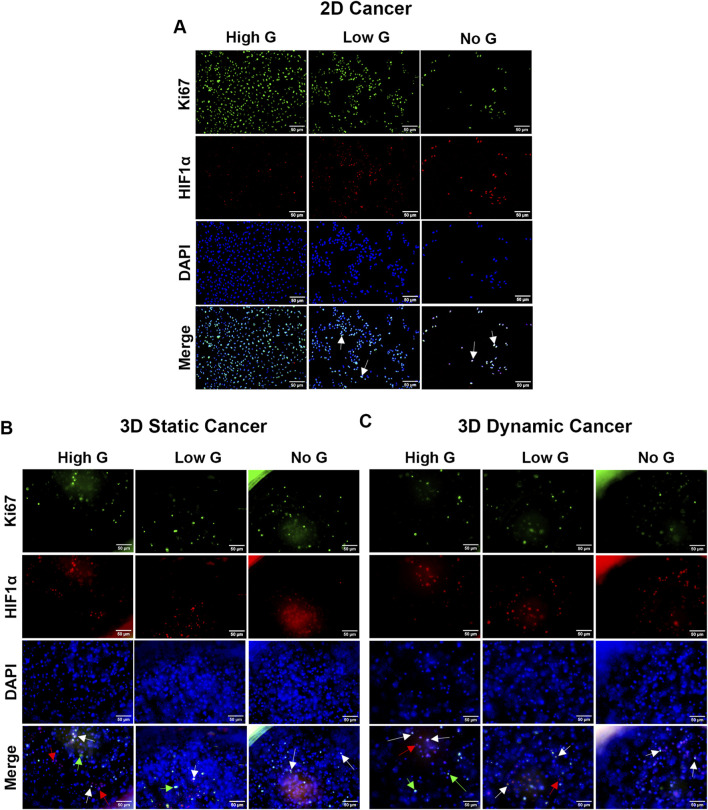
Cancer cells proliferation and hypoxia. Representative images of immunostaining of ki67 and HIF1α on 2D static **(A)** 3D static **(B)** and 3D dynamic **(C)** cancer models. Pictures show Ki67 (green), HIF-1α (red), and nuclei (DAPI, blue). Merged images highlight proliferating cells (green arrows), hypoxic cells (red arrows), and cells co-expressing both markers (white arrows). Scale bar: 50 μm.

Since cancer cells predominantly rely on aerobic glycolysis for energy production, glucose deprivation is expected to induce cellular stress responses that may affect proliferation and survival (Pan et al., 2025; Han et al., 2015). To further investigate these effects, cancer cell proliferation and stress-related pathways were evaluated by immunostaining for Ki67 and HIF-1α (Figure 3).

In 2D cultures, the percentage of Ki67^+^ cells remained relatively high across all glucose conditions, despite an overall reduction in cell number under Low G and No G conditions, suggesting that the surviving cell population retains proliferative potential.

HIF-1α signal was detected across all conditions and appeared more evident under Low G and No G conditions compared to High G. Although HIF-1α is typically associated with hypoxic responses, its presence under normoxic, glucose-deprived conditions may reflect stabilization driven by metabolic stress pathways rather than hypoxia *per se*, as previously reported in glucose-deprived contexts where pseudo-hypoxic signaling can occur.

Interestingly, co-localization of Ki67 and HIF-1α was observed, particularly under No G conditions. Rather than indicating active proliferation under stress, this may suggest that a subset of cells maintains cell-cycle activity while engaging stress-response pathways, potentially reflecting a heterogeneous adaptive response to glucose deprivation.

In 3D static and dynamic models, both Ki67 and HIF-1α signals were detectable under all conditions. Co-expression of the two markers was also observed, although less prominently than in 2D cultures. This attenuated response may be associated with the structural and diffusional properties of the 3D microenvironment, which can modulate nutrient gradients and cellular stress responses.

Overall, glucose deprivation influenced both proliferation- and stress-related markers in 2D and 3D systems, with the most evident changes observed under complete glucose deprivation. These findings suggest a complex and heterogeneous cellular response to metabolic stress rather than a uniform shift in proliferative or hypoxic behavior.

### 2.4 Glucose deprivation partially impaired healthy cell viability in 3D *in vitro* model

Fasting and caloric restriction have been widely studied in tumor cells, but their impact on non-pathological tissues remains less understood. To explore this, human dermal fibroblasts (HDFas), used here as a representative non-cancer stromal cell model, were cultured in 2D static, 3D static, and 3D dynamic models under High G, Low G, or No G conditions. After 3 days, cell morphology, viability and AlamarBlue signal were assessed to determine the impact of glucose modulation on healthy tissues.

**FIGURE 4:**
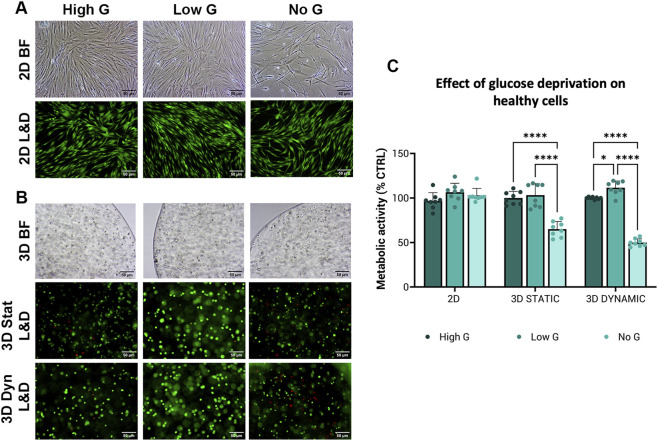
Effects of glucose deprivation on healthy cell viability and metabolic activity. Representative images in BF and Live/Dead staining of HDFs cells in 2D **(A)** and 3D **(B)** conditions cultured in High glucose (High G), Low glucose (Low G) or No glucose (No G) containing media for 3 days. Scale bar: 50 μm **(C)** Metabolic activity assessed by AlamarBlue assay. Semi-quantitative data are reported as mean ± SD and expressed as percentage of control (High G). Statistical comparison was made using two-way ANOVA (N = 3), *p < 0.05, ****p < 0.0001.

In 2D cultures, fibroblasts maintained their typical spindle-shaped morphology and high confluency across all glucose conditions, including No G, with no evident rounding, detachment, or cell death (Figure 4A). In 3D cultures (Figure 4B), Live/Dead staining revealed a homogeneous distribution of viable cells across both static and dynamic models. A modest increase in red fluorescent signals was detected in No G groups, suggesting a mild induction of cell death under full glucose deprivation.

These qualitative findings were consistent with the semi-quantitative data obtained by AlamarBlue assay (Figure 4C). Unlike cancer cells, 2D cultured fibroblasts maintained comparable metabolic activity across all glucose conditions. In contrast, both static and dynamic 3D cultures displayed a statistically significant reduction in viability-related metabolic activity in No G conditions compared to their respective High G controls (p < 0.0001), while Low G had no significant effect. This suggests that moderate glucose restriction is well tolerated, while complete glucose deprivation partially compromises cell function, particularly in 3D environments that better reflect tissue complexity.

Collectively, healthy fibroblasts remain largely resilient to moderate glucose reduction, but severe deprivation reduces cell activity in physiologically relevant 3D models, highlighting potential vulnerability of normal tissues under extreme glucose restriction.

### 2.5 Combined effects of chemotherapy and glucose-deprivation in breast cancer models

To investigate the combined effects of chemotherapy and nutrient modulation, the interaction between cisplatin and glucose deprivation (used as an extreme stress condition) was evaluated in 2D, 3D static, and 3D dynamic breast cancer models. Importantly, since reduced glucose availability (Low G) did not impair viability in any of the previously tested models, only full glucose removal (No G, named as glucose deprivation) was considered for combination with drug treatment.

A two-step treatment strategy was adopted: cells were first pre-conditioned for 24 h either in glucose-rich (G^+^) or glucose-free (G^−^) medium, then treated with cisplatin in either presence (G^+^) or absence (G^−^) of glucose, finally generating four combinations (i.e., G^+^G^+^, G^+^G^−^, G^−^G^+^, G^−^G^-^) plus untreated controls (G^+^ and G^−^), reflecting all possible interactions between nutrient availability and chemotherapy exposure.

**FIGURE 5:**
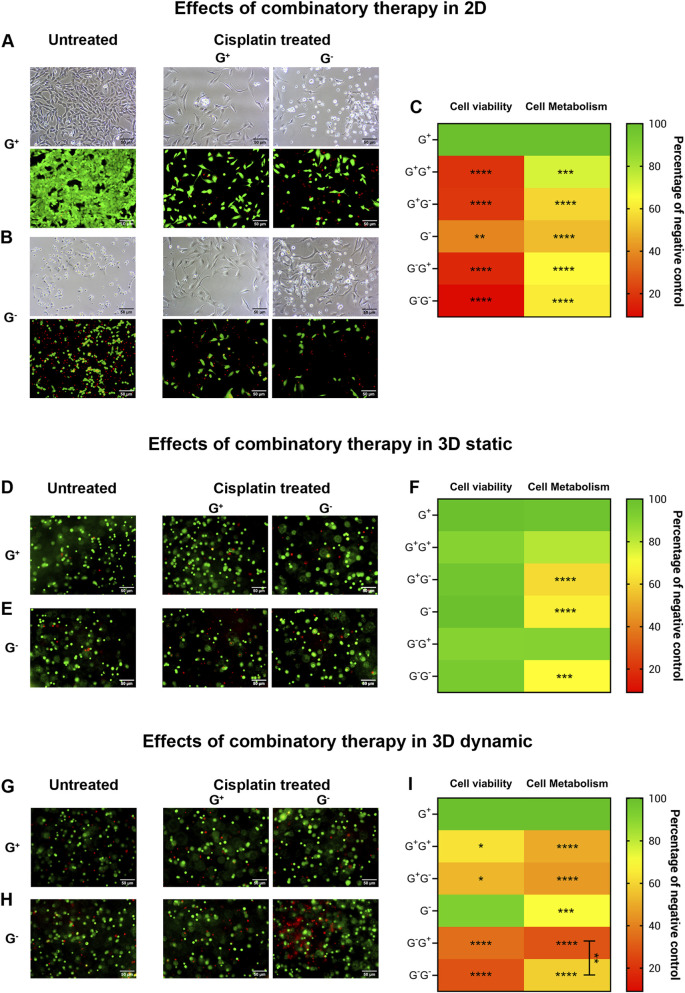
Effects of different glucose deprivation adjuvating chemotherapy treatment strategies on tumor models. Representative images of MDA-MB-231 cancer cells and Live/Dead staining after either glucose-rich medium or glucose-free medium pre-conditioning in 2D **(A,B)**, respectively) 3D static **(D,E)**, respectively and 3D dynamic **(G,H)**, respectively conditions under different drug treatment. Scale bar: 50 μm. Heatmap showing semi-quantitative analysis on cell metabolism by AlamarBlue assay and cell viability from Live/Dead images in 2D **(C)**, 3D static **(F)** and 3D dynamic **(I)** conditions. Semi-quantitative data are reported as mean ± SD and expressed as percentage of control (High G, No drug), *p < 0.05, **p < 0.01, ***p < 0.001, ****p < 0.0001 show comparison with negative control, according to two-way ANOVA, N = 5 independent experiments. G^+^: glucose-rich medium; G^−^: glucose-free medium.

2D cultured preconditioned with glucose-rich medium revealed a marked reduction in both cell number and viability after drug administration (G^+^G^+^, G^+^G^−^) compared to the untreated samples (Figure 5A). Interestingly, glucose deprivation applied only during drug exposure (G^+^G^−^) did not visibly enhance the cytotoxic response compared to standard treatment conditions (G^+^G^+^), suggesting that short-term glucose deprivation applied only during drug exposure may not be sufficient to enhance cisplatin sensitivity in 2D cultures.

By contrast, pre-conditioning in glucose-free medium (Figure 5B), increased baseline cell stress (G^−^), and subsequent cisplatin exposure (G^−^G^+^ and G^−^G^-^) further reduced cell viability, showing enhanced cell death compared to their counterparts pre-conditioned in glucose-rich medium (G^+^G^+^, G^+^G^−^). Quantitative analyses of Live/Dead and AlamarBlue assays were consistent with these observations, although differences between treatment groups did not reach statistical significance (Figure 5C).

In the 3D Static model, Live/Dead imaging showed high viability across all treatments (Figures 5D,E). Quantitative analysis confirmed no significant reduction in viability compared to untreated samples (Figure 5F). However, a significant reduction in metabolic activity was observed compared to the negative control when glucose was removed during treatment (Figure 5F), indicating a functional response to nutrient restriction despite limited overt cytotoxicity. Moreover, G^−^ pre-conditioning followed by cisplatin exposure in glucose-rich conditions (G^−^G^+^), was associated with partial recovery of the ability to reduce AlamarBlue reagent. Since viability remained largely unchanged across conditions, these variations likely reflect differences in the overall functional state of the cultures rather than major changes in cell survival. Indeed, from a diffusion standpoint, glucose may readily penetrate extracellular matrices due to its small size. In contrast, the limited response to cisplatin under static conditions may be attributed to the difference between the nominal bulk concentration and the effective concentration reaching the cells. In static 3D environments, the absence of fluid motion leads to a rapid depletion of the drug at the gel periphery and an accumulation of dead cells and debris, which significantly increase the mass-transfer resistance and limit the drug’s inward diffusion.

In the 3D Dynamic model, a clear increase in dead cells was visible across all drug-treated groups (Figures 5G,H), accompanied by a significant reduction in cell viability (Figure 5I), demonstrating effective drug penetration under fluid flow conditions. Unlike static systems, the continuous perfusion in the cancer on chip platform maintains a constant concentration gradient and facilitates the removal of cellular debris, thereby reducing the resistance to cisplatin diffusion and ensuring that the effective concentration within the tumor construct reaches therapeutically relevant levels. This condtion allowed to clearly distinguish the influence of nutrient conditions: glucose-free pre-conditioning alone (G^−^), significantly reduced viability-related metabolic activity (p < 0.001 compared to negative control) but did not affect viability, suggesting a functional impact of nutrient restriction preceding overt cell death. Moreover, when cisplatin was administered after glucose-free pre-conditioning (G^−^G^+^ and G^−^G^-^), viability dropped sharply regardless of the glucose level during treatment, and this reduction was more pronounced than in the High-G pre-conditioned counterparts (G^+^G^+^, G^+^G^−^). Interestingly, when glucose was reintroduced during cisplatin exposure (G^−^G^+^), metabolic activity was significantly more impaired than in the full glucose-free condition (G^−^G^-^) (p < 0.01).

This observation may suggest that dynamic changes in nutrient availability influence cellular response to chemotherapy, potentially reflecting transient metabolic vulnerabilities rather than stable adaptive states.

Overall, these findings indicate that nutrient pre-conditioning and culture dynamics modulate the cellular response to chemotherapy, with dynamic 3D systems enabling the detection of context-dependent treatment effects that are less evident in static or 2D conditions.

### 2.6 Effects of combined glucose deprivation and chemotherapy in healthy fibroblasts

To estimate how non-cancerous cells respond to combined nutritional and chemotherapeutic challenges, HDFas were cultured in 3D dynamic systems, replicating the treatment design applied for TNBC cells.

**FIGURE 6:**
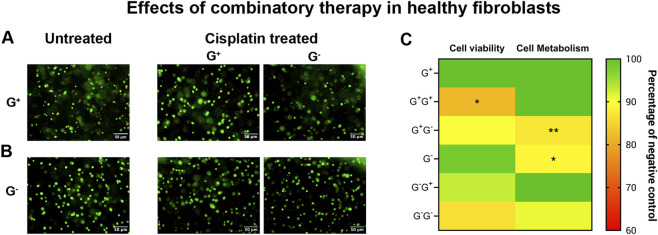
Effects of different glucose deprivation adjuvating chemotherapy treatment strategies on healthy models. Representative images of Live/Dead staining in 3D dynamic conditions after either glucose-rich **(A)** or glucose-free **(B)** medium pre-conditioning and under drug treatment. Scale bar: 50 μm. **(C)** Heatmap showing semi-quantitative analysis on cell metabolism by AlamarBlue assay and cell viability from Live/Dead images in 3D dynamic conditions. Semi-quantitative data are reported as mean ± SD and expressed as percentage of Control (High G, No drug), *p < 0.05, **p < 0.01, ****p < 0.0001 show comparison with negative control, according to two-way ANOVA, N = 2 independent experiments.

Under standard conditions (G^+^G^+^), cisplatin treatment induced a modest but significant reduction in fibroblast viability compared to untreated controls (p < 0.05), consistent with the known cytotoxic effects of cisplatin on non-cancer cells (Figures 6A–C). Glucose deprivation alone (G^−^) did not significantly affect cell viability but was associated with a reduction in metabolic activity, indicating that fibroblasts can maintain survival under nutrient stress despite functional alterations.

Interestingly, when combined with cisplatin, glucose deprivation produced sequence-dependent effects. In particular, glucose deprivation applied during the pre-conditioning phase (G^−^G^+^ and G^−^G^-^) was associated with a modest attenuation of cisplatin-induced cytotoxicity compared to standard treatment conditions (G^+^G^+^) (Figures 6B,C). As expected, metabolic activity was reduced under glucose-free conditions and showed a slight recovery upon glucose reintroduction (G^−^G^+^), reflecting the dependence of this readout on nutrient availability.

Overall, these findings underscore the relevance of dynamic 3D models in revealing differential treatment responses. However, these effects were limited and not consistently observed across all readouts, indicating a mild and context-dependent response that warrants further investigation.

### 2.7 Dynamic 3D cancer model shows qualitative concordance with clinical treatment patterns

**FIGURE 7:**
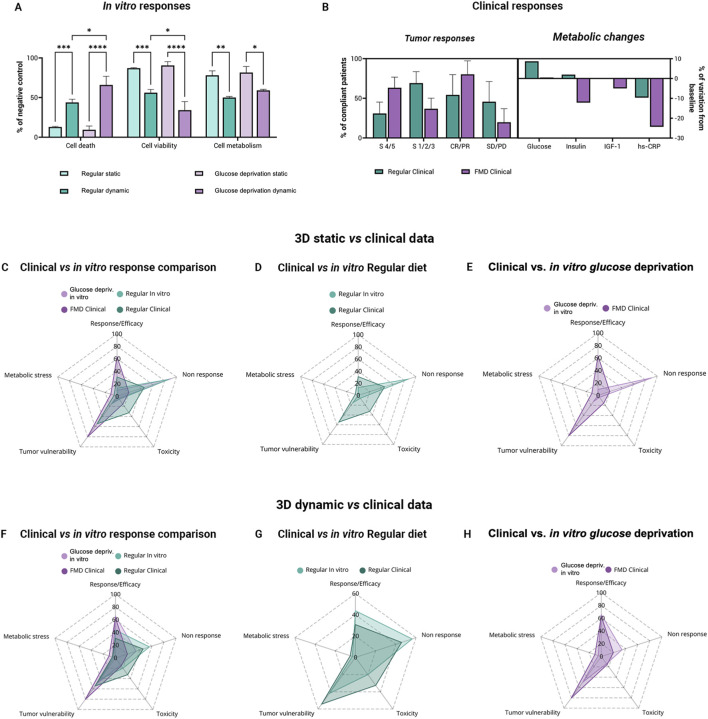
Qualitative comparison of *in vitro* and clinical responses under regular and glucose deprivation conditions. **(A)** Bar plots showing *in vitro* responses as the percentage variation (vs. negative control) of cell death, cell viability, and cell metabolism in tumor cells cultured in 3D static or dynamic conditions and treated with either conventional therapy (Regular, High G pre-conditioning + High G drug) or glucose deprivation (glucose deprivation pre-conditioning + drug with glucose deprivation). Data are reported as mean ± S.D. *p < 0.05, **p < 0.01, ***p < 0.001, ****p < 0.0001, according to two-way ANOVA. **(B)** Bar blot showing clinical responses. Left: distribution of tumor responses in patients receiving standard chemotherapy under Regular diet or FMD, classified as Miller–Payne grades (4/5 = responders; 1/2/3 = partial or minimal response) and RECIST outcomes (CR/PR = responders; SD/PD = non-responders). Right: systemic metabolic variations (glucose, insulin, IGF-1, hs-CRP) expressed as percentage change from baseline in Regular vs. FMD patients. **(C)** Radar plot showing clinical vs*. in vitro* 3D static response comparison. Overlays show Regular vs. FMD conditions in clinical data and *in vitro* 3D static cultures. **(D)** Radar plot comparing only Regular diet clinical data with Regular diet 3D static *in vitro* responses. **(E)** Radar plot comparing only FMD clinical data with glucose deprivation 3D static *in vitro* responses. **(F)** Radar plot showing clinical vs*. in vitro* 3D dynamic response comparison. **(G)** Radar plot comparing Regular diet clinical data with Regular diet 3D dynamic *in vitro* responses. **(H)** Radar plot comparing FMD clinical responses with glucose deprivation 3D dynamic *in vitro* responses. Radar plots integrate the five comparative domains: efficacy/response (cell death - MP 4/5), non-response (cell viability - SD/PD), tumor vulnerability (metabolic reduction - CR/PR), toxicity (death in healthy cells - clinical toxicity score), and metabolic stress (stress in healthy cells - systemic metabolic changes).

Effective translation of preclinical findings requires models that capture key features of patient responses. To determine whether our *in vitro* CoC model reflects clinically observed trends, an exploratory qualitative comparison was performed between *in vitro* readouts (Figure 7A) and clinical outcomes derived from two published trials in HER2-negative stage II/III breast cancer patients undergoing chemotherapy with or without a fasting-mimicking diet (FMD) (Figure 7B) (de Groot et al., 2020; Bahrami et al., 2024). Clinical endpoints included pathological response (Miller and Payne grading) and radiological response (RECIST criteria) (Table 1).

**TABLE 1:** Efficacy of chemotherapy based on pathological and radiological tumor response data (De Groot et al., 2020, Bahrami et al., 2024).

Clinical parameter	Regular diet			FMD^a^		
​	Trial 1	Trial 2	Av. ± SD	Trial 1	Trial 2	Av. ± SD
Score 4/5 Miller and Paine	41%	20.7%	30.8% ± 14.3%	72.7	53.8	63.2 ± 13.3
Score 1/2/3 Miller and Paine	59%	79.3%	69.1% ± 14.3%	27.3	46.2	36.8% ± 13.3%
CR/PR^b^	36.3%	72.3%	54.3% ± 25.4%	68.1	92.3	80.2% ± 17.1%
SD/PD^c^	63.7%	27.7%	45.7% ± 25.4%	31.9	7.7	19.8% ± 17.1%

^a^ Fasting-Mimicking Diet.

^b^ Complete Response/Partial Response, radiological responders.

^c^ Stable Disease/Progressive Disease, radiological non-responders.

The Miller and Payne grades is a 5-grade scale used in breast cancer pathology to assess how well a tumor responds to chemotherapy: patients with high score (4/5) exhibit a marked loss of viable tumor cells (≥ 90%) in surgical specimens and are therefore classified as responders, whereas lower grades (1–3) indicate only partial or minimal tumor-cell reduction and patients are typically categorized as non-responders (Ogston et al., 2003; Wang et al., 2021). On the other side, Response Evaluation Criteria in Solid Tumors (RECIST) provide a standardized set of rules to determine whether solid tumors improve (respond), remain stable, or progress during treatment, based on quantitative radiological assessment of target and non-target lesions. According to RECIST, Complete Response (CR) corresponds to the disappearance of all target lesions, while Partial Response (PR) indicates a ≥ 30% reduction in the sum of target lesion diameters. Stable Disease (SD) reflects changes that do not meet the criteria for either response or progression, whereas Progressive Disease (PD) denotes a ≥ 20% increase in target lesion size or the appearance of new lesions.

To enable the comparison, selected *in vitro* parameters were considered as approximation of clinical endpoints. Conventional therapy performed under regular-glucose conditions (G^+^G^+^) was compared with the Regular diet, while full glucose deprivation conditions (G^−^G^-^) were compared with FMD. Tumor cell death was associated with pathological response, while cell viability and metabolic activity were used to represent features related to treatment resistance and early tumor adaptation. Similarly, changes observed in healthy cells were used as an approximation of treatment-related toxicity (Table 2) and systemic metabolic perturbation (Table 3). These associations do not represent direct equivalences but provide a framework to compare overall response patterns across systems.

**TABLE 2:** Grade III toxicity during chemotherapy between Regular diet and FMD patient groups (Bahrami et al., 2024).

Clinical parameter	Regular diet	FMD^a^
Diarrhea	22.7%	13.6%
Vomiting	54.5%	13.6%
Nausea	40.9%	18.2%
Constipation	13.6%	4.5%
Neutropenia	45.5%	22.7%
Thrombocytosis	4.5%	4.5%
Mouth sores	45.5%	31.8%
Av.	33.3%	15.56%

^a^ Fasting-Mimicking Diet.

**TABLE 3:** Score of variation of metabolic and anthropometric parameters between Regular diet and FMD groups after chemotherapy (Bahrami et al., 2024).

Clinical parameter	Regular diet			FMD^a^		
​	Baseline	After chemo	% Of variation	Baseline	After chemo	% Of variation
Glucose	97.50	106.00	8.72%	97	97.50	0.52%
Insulin	12.60	12.85	1.98%	14	12.30	−12.14%
IGF-1	181.00	181.00	0.00%	181	172.00	−4.97%
hs-CRP	5.70	5.15	9.65%	4.61	3.49	−24.30%
Av.	​	​	5.09%	​	​	−10.22%

^a^ Fasting-Mimicking Diet.

When comparing model outputs, the 3D static system showed limited correspondence with clinical trends. Radar plots (Figures 7C–E) revealed inconsistencies across response categories and an inability to clearly distinguish between regular diet and FMD conditions.

In contrast, exhibited a closer qualitative alignment with clinical patterns (Figures 7F-H). In particular, the dynamic system captured the relative differences between treatment conditions across multiple response dimensions, including efficacy-related and metabolic parameters. While not predictive in a quantitative sense, this concordance suggests that dynamic culture conditions may better reflect the integrated and context-dependent responses observed in patients.

Importantly, these observations should be interpreted cautiously. The present *in vitro* system does not reproduce the systemic endocrine, immune, and metabolic complexity of fasting-mimicking interventions in patients, and the comparison is not intended to establish predictive or quantitative clinical correlations. Rather, this exploratory analysis suggests that dynamic culture conditions may improve the ability of *in vitro* dynamic models to capture the *in vivo* context-dependent treatment-response patterns compared to static systems.

Overall, these findings support the potential utility of dynamic 3D cancer-on-chip systems as complementary mechanistic tools for investigating nutrient-dependent modulation of chemotherapy responses.

## 3 Discussion

Metabolic plasticity profoundly influences cancer sensitivity to chemotherapy, and the tumor microenvironment plays a central role in shaping this response (Fendt et al., 2020). Fasting‐mimicking strategies have therefore attracted interest as adjuvant approaches to enhance treatment efficacy while reducing systemic toxicity. However, their translation into preclinical settings remains challenging. Traditional 2D cultures fail to reproduce key features of solid tumors, such as nutrient gradients, extracellular matrix interactions, and dynamic transport that characterize solid tumors *in vivo*, often leading to discrepancies between *in vitro* observations and clinical outcomes. On the other side, preclinical animal models are poorly suited for studying fasting-based anticancer therapies due to major interspecies differences in metabolism and systemic stress responses (Fazeli and Steinhauser, 2025).

Although clinical fasting and fasting-mimicking interventions involve systemic metabolic adaptations extending beyond glucose availability alone, extracellular glucose modulation represents one of the most experimentally controllable nutrient-related parameters in simplified *in vitro* systems.

In this context, we developed and evaluated a set of complementary *in vitro* models integrating metabolic modulation and chemotherapy across increasing levels of microenvironmental complexity, including 2D monolayers, 3D static constructs, and perfused 3D organ-on-chip systems. These models were further compared, in a qualitative manner, with clinical data from patients undergoing chemotherapy with or without fasting-mimicking dietary regimens, with the aim of assessing whether key response patterns could be recapitulated *in vitro*.

Our data showed a clear dichotomy between modest glucose reduction and complete glucose deprivation. A mild lowering of extracellular glucose (Low G) did not significantly impair viability, consistent with the well-documented metabolic plasticity of many cancer cells, which can rapidly compensate for restricted glycolytic flux through upregulation of alternative pathways such as oxidative phosphorylation, fatty acid oxidation or autophagy-mediated recycling of intracellular substrates (Zheng, 2012; Nam et al., 2024). In contrast, full glucose withdrawal (No G) consistently produced a dramatic loss of metabolic activity and increased cell death, in agreement with previous reports linking glycolytic input removal to ATP and redox failure, with consequent activation of stress and cell-death pathways (Kang et al., 2023; Khajah et al., 2022; Mathews et al., 2014).

Importantly, cellular responses were strongly influenced by the culture system. Both static and dynamic 3D constructs were less sensitive to glucose deprivation than 2D monolayers, consistent with the greater resistance of 3D systems due to matrix embedding and diffusion gradients (Fontoura et al., 2020; Kapałczyńska et al., 2016). These features contribute to a more heterogeneous and buffered response to metabolic stress, suggesting that 3D systems provide a more realistic estimate of treatment effects compared to 2D monolayers.

Analysis of Ki67 and HIF-1α further supported the complexity of cellular adaptation to metabolic stress. While Ki67 positivity remained relatively preserved, HIF-1α signal was detected under glucose-restricted conditions, suggesting the engagement of stress-related pathways (De Berardinis and Chandel, 2016; Eales et al., 2016). Multiple clinical studies have reported that proliferative indices, including Ki67, do not necessarily decline during short-term fasting or nutrient restriction, suggesting that tumor cells can maintain growth despite reduced glucose availability (Safdie et al., 2009; de Groot et al., 2015). On the other side, although HIF-1α is classically associated with hypoxia, its presence under normoxic, glucose-deprived conditions may reflect non-canonical stabilization linked to metabolic stress (Rohwer and Cramer, 2011; Keith et al., 2011). Finally, The observation of Ki67/HIF-1α double-positive cells may therefore indicate a heterogeneous cellular response, in which a subset of cells maintains proliferative activity while engaging stress-response pathways. Several clinical and *in vivo* studies associated this phenotype with enhanced aggressiveness, metabolic flexibility, and resistance to therapy (Schito and Semenza, 2016). However, the attenuated HIF-1α response in 3D cultures is consistent with evidence that 3D architectures buffer acute metabolic shocks through matrix interactions, altered diffusion profiles, and slower proliferation dynamics (Friedl and Alexander, 2011; Edmondson et al., 2014), thereby moderating the stress signature observed in 2D and reinforcing their greater physiological relevance.

Building on the observations from nutrient-only treatments, the introduction of cisplatin into the experimental design allowed us to assess how metabolic state modifies chemotherapeutic responses in models of increasing microenvironmental complexity. The most striking evidence emerged from the comparison between static and dynamic 3D conditions: while static 3D cultures showed minimal cytotoxic response to cisplatin, dynamic 3D models displayed clear drug-induced cell death, confirming that flow is essential for efficient drug penetration and distribution within 3D tissues (Marrella et al., 2021; Zhuang et al., 2019). A critical factor in interpreting the disparate responses between our 2D, 3D static, and 3D dynamic models is the concept of effective concentration. As highlighted in recent simulation studies (Marrella et al., 2021), the actual concentration of a drug reaching a cell in a 3D construct can differ significantly from the nominal concentration in the bulk medium due to transport limitations. In our findings, the high resistance to cisplatin in static 3D models, contrasted with the high sensitivity in dynamic models, aligns with the understanding that fluid flow modulates the mass transport of solutes. Specifically, while static systems may initially present high gradients, the lack of convection and the buildup of a “barrier” of dead cells hinder deep penetration of the drug. Conversely, the MIVO® platform’s perfusion system maintains a steady-state delivery and effectively “clears” the microenvironment, allowing the effective concentration of cisplatin to mirror the intended therapeutic dose more closely than in static 3D or 2D setups, where the biological context of transport is either absent.

In addition, the timing of glucose deprivation played an important role. Interestingly, while glucose restriction applied only during drug treatment did not enhance cisplatin efficacy in 2D models and showed variable effects in 3D systems, pre-conditioning under glucose deprivation was associated with a tendency toward increased sensitivity to cisplatin, according with studies demonstrating that FMD can sensitize cancer cells to chemotherapy (Lee et al., 2012; Brandhorst, 2021; Kikomeko et al., 2023; Caffa et al., 2020). Moreover, only dynamic culture allowed to appreciate that reintroduction of glucose after deprivation (“glucose OFF → glucose ON” condition), was associated with a further reduction in AlamarBlue signal, suggesting persistent functional impairment following glucose reintroduction rather than immediate recovery. Although this phenomenon requires deeper mechanistic investigation, it highlights the importance of temporal aspects of metabolic interventions.

The behavior of healthy fibroblasts provides an important internal control for differential stress resistance. In dynamic 3D culture, conventional chemotherapy reduced fibroblast viability, as expected for an off-target cytotoxic effect, but glucose-free preconditioning clearly mitigated these effects, although impairing metabolic function. This selective protection of healthy cells has been widely reported in fasting/FMD literature (Raffaghello et al., 2010; Raffaghello et al., 2008) and strengthens the translational relevance of metabolic preconditioning strategies.

Collectively, these data indicate that metabolic preconditioning shapes chemotherapeutic responses in a coordinated, tissue-specific manner, setting the stage for evaluating whether these *in vitro* patterns mirror the systemic effects observed in patients. To explore the potential translational relevance of these findings, we performed a qualitative comparison between *in vitro* results and clinical data from fasting-mimicking diet trials. While no quantitative prediction can be inferred, the 3D dynamic model showed a closer alignment with clinical response patterns compared to static systems. This observation suggests that incorporating dynamic culture conditions may improve the ability of *in vitro* platforms to capture integrated and context-dependent treatment responses observed in patients. Although clinical trials of fasting/FMS remain limited and larger studies are needed (Maes et al., 2025), the agreement between patient trends and our *in vitro* results supports the translational relevance of the fasting-mimicking CoC platform. This aligns with extensive literature demonstrating that dynamic flow improves nutrient and oxygen transport, maintains physiological gradients, and enhances metabolic realism, enhancing the predictive power of tumor models (Sontheimer-Phelps et al., 2019; Wan et al., 2021; Kim et al., 2025).

Despite the coherence of our findings with published clinical data, some limitations must be acknowledged. The study primarily relies on viability, AlamarBlue assay, and selected stress markers, without detailed characterization of underlying metabolic pathways or signaling networks. Importantly, the present platform should be considered a reductionist tumor-centered model designed to isolate nutrient-dependent effects within a controlled microenvironment, rather than a comprehensive representation of systemic fasting physiology. The clinical comparison is based on indirect associations and extreme *in vitro* conditions (complete glucose deprivation) that do not fully mirror the physiological glucose levels maintained in patients during FMD. Therefore, results should be interpreted as qualitative trends rather than quantitative predictions. Furthermore, the use of a single cancer cell line (MDA-MB-231) without taking into account additional TNBC models, and skin-derived fibroblasts (HDFas) instead of breast-specific normal cells limits the generalizability of the toxicity findings. Future studies integrating multiple cell types, patient-derived models, and pathway-level analyses will be necessary to further validate and extend these observations.

Overall, our findings support the growing consensus that dynamic flow is not an optional refinement but a prerequisite for translational relevance. Dynamic 3D cultures uniquely captured the multidimensional clinical signatures of chemotherapy with or without fasting-mimicking conditions, underscoring their value as next-generation preclinical platforms capable of anticipating patient responses.

## 4 Methods

### 4.1 Cell cultures

MDA-MB-231 (ATCC®, Manassas, VA, United States, human triple-negative breast cancer cell line) and HDFas cells (CELLnTEC, Bern, Swiss, normal human dermal fibroblasts used as healthy tissue controls) were cultured in Dulbecco’s Modified Eagle Medium (DMEM) high glucose (Thermofisher scientific, Waltham, MA, United States) supplemented with 10% fetal bovine serum (FBS, Euroclone, Milan, Italy), 1% penicillin/streptomycin (Euroclone), and 2 mM L-glutamine (Euroclone) (complete medium). Cells were expanded under standard conditions (37 °C, 5% CO_2_) and passaged at 70%–90% confluence.

For subsequent experiments, cells were either cultured in two-dimensional (2D) or three-dimensional (3D) culture conditions. Two-dimensional models were established by seeding 1.5 × 10^4^ cells (MDA-MB-231 or HDFas) into each well of a 24-well plate. Cells were incubated for 24 h at 37 °C and 5% CO_2_, to allow attachment, prior to be used for the experiments. For all the analyses, passage 5 cells were used.

### 4.2 Three-dimensional cell culture model

Three-dimensional (3D) tumor and healthy tissue models were established through an alginate-based hydrogel. Cells were resuspended in complete medium at a concentration of 1.5 × 10^6^ cells/mL and mixed 1:1 (*v/v*) with a sterile 2% (*w/v*) sodium alginate solution (React4Life S. p.A., Genoa, Italy) in 0.9% NaCl (Ecotainer®, B. Braun, Milan, Italy), resulting in a final 1% (*w/v*) suspension containing 7.5 × 10^5^ cells/mL. Spherical hydrogels embedding 1.5 × 10^4^ cells were obtained through physical crosslinking by dropping 20 μL of alginate-based cell suspension onto 0.5 M CaCl_2_ sterile solution. After gelation, 3D cell-laden hydrogels were washed in 0.9% NaCl and cultured for 24 h in complete medium supplemented with 5 mM CaCl_2_ before treatment.

### 4.3 Dynamic culture in MIVO® cancer-on-chip

Physiological-like perfusion was reproduced using the MIVO® Single Flow organ-on-chip device (React4life), that is a milli-fluidic PDMS-free device for 3D fluid-dynamic cell culture. The system was assembled under sterile conditions and connected to a peristaltic pump (RJ100, React4life) to create a closed-loop circuit. Each MIVO® circuit was filled with 3.5 mL of culture medium in the basal compartment. Two 3D tumor constructs were transferred into a 24-well cell culture insert (8 µm pore size, Greiner Bio-One GmbH, Austria), pre-filled with 250 µL of medium, and then moved into the MIVO® chamber. The assembled system was placed in incubator at 37 °C and 5% CO_2_. A constant velocity of 0.37 mm/s was applied (i.e., 1 mL/min flow rate), allowing continuous medium circulation and dynamic culture conditions throughout the experimental period.

### 4.4 Glucose restriction simulation

The effect of glucose availability on tumor and healthy *in vitro* models was assessed by culturing the systems under different glucose conditions for 3 days. The experimental setups included: High glucose (4.5 g/L D-glucose), Low glucose (1 g/L D-glucose), and No glucose (0 g/L D-glucose) included in the culture media, both in the apical and basal compartment. Media formulations were based on commercial DMEM (Gibco™, Waltham, MA, United States) supplemented with 10% fetal bovine serum (FBS), 1% penicillin/streptomycin, and 5 mM CaCl_2_ to maintain alginate gel stability in 3D models. After a 24-h pre-culture period, each system (2D, 3D static, 3D dynamic) was treated with the three different glucose media for 72 h. Following treatment, cell viability and metabolism were assessed.

### 4.5 Drug treatment

The *in vitro* models (2D, 3D static, and 3D dynamic) obtained as previously described were subjected to a two-step protocol to assess the effects of cisplatin chemotherapy placed in circulation to resemble the intravenous administration, either alone or in combination with glucose deprivation. In the first phase (pre-conditioning), cells were pre-conditioned for 24 h in either high-glucose medium (4.5 g/L D-glucose; High glucose pre-conditioning) or glucose-free medium (0 g/L D-glucose; glucose deprivation pre-conditioning). In the second phase (treatment), the medium was replaced after washing, and cells were cultured for an additional 48 h under three conditions: without drug, with 10 µM cisplatin in High glucose, or with 10 µM cisplatin in No glucose conditions. This two-step design resulted in six experimental conditions, obtained by the combination of the two pre-conditioning media and the three treatment conditions.

### 4.6 Cell metabolism assessment: AlamarBlue assay

A semi-quantitative analysis of cellular metabolic activity was performed by using the AlamarBlue assay (Thermofisher scientific), following the manufacturer’s instructions. Briefly, hydrogels from 3D static and 3D dynamic models were aseptically transferred into individual wells of a 24-well plate using sterile tweezers. Both 2D cultured cells and 3D models were incubated with fresh media containing 10% *v/v* AlamarBlue reagent for 3 h and 6 h, respectively. A blank control (without cells) was prepared under the same conditions. After incubation, 100 µL of the reduced reagent from each well was collected (four replicates per sample) and transferred into a 96-well flat-bottom plate (VWR). Absorbance was measured using a microplate spectrophotometer (Infinite® M Nano, Tecan) with i-control™ 2.0 software. To prevent photodegradation of the reagent, light exposure was minimized during all handling steps.

### 4.7 Cell viability assessment: live/dead assay

A qualitative analysis of cells viability was also performed by using a commercial Live and Dead fluorescence-based viability assay (ab115347, Abcam Limited, United States). The working solution was prepared following the manufacturer’s instructions by diluting the Live Cell Staining Dye (2 μL/mL) and Dead Cell Staining Dye (1 μL/mL) in the appropriate assay buffer. Both 2D cultured cells and 3D models were incubated for 15 min in staining solution and then fluorescence images were acquired using an inverted fluorescence microscope (Nikon Eclipse Ts2-FL). Number of live and dead cells was quantified in at least three independent area, and viability was expressed as the percentage of live cells per ROI. These values were subsequently normalized to the corresponding negative control (High G, No drug).

### 4.8 Immunofluorescence staining

Immunofluorescence staining was performed to assess the expression of proliferation and hypoxia markers, Ki67 and HIF-1α, in both 2D and 3D models. Samples were fixed with 4% formaldehyde at room temperature for 15 min and cells were permeabilized using 0.1% Triton X-100 in NaCl/CaCl_2_ for either 15 min (2D) or 2 h (3D). Blocking was performed with 2% BSA for 1 h (2D) or 2 h (3D). Samples were then incubated overnight with a mix of primary antibodies: rabbit anti-human Ki67 (1:1000, Abcam ab15580) and mouse anti-human HIF-1α (1:200, Abcam ab1), diluted in 0.2% BSA in NaCl/CaCl_2_. The following day, after three washes, secondary antibodies (goat anti-rabbit AlexaFluor488 and goat anti-mouse AlexaFluor555, both 1:200, diluted in 0.2% BSA in NaCl/CaCl_2_) were applied for 1 h (2D) or 2 h (3D) at room temperature in the dark. Finally, nuclei were counterstained with 1 µM DAPI (Thermo Fisher Scientific) for 15 min (2D) or 30 min (3D). After final washes, 3D hydrogels were transferred onto coverslips to preserve structure, and all samples were imaged using an inverted fluorescence microscope (Nikon Eclipse Ts2-FL).

### 4.9 Clinical-*in vitro* correlation analysis

Publicly available clinical response data were extracted from two published clinical trials (de Groot et al., 2020; Bahrami et al., 2024) evaluating the effects of chemotherapy administered under either a regular diet or a fasting-mimicking diet (FMD). From these studies (i) pathological response categories (Miller and Payne grades 1–5), grouped into high responders (score 4/5) and low-to-intermediate responders (score 1/2/3); (ii) radiological response outcomes (Complete Response CR, Partial Response PR, Stable Disease SD, Progressive Disease PD); and (iii) metabolic and toxicity parameters were collected. To generate composite indices, metabolic biomarkers (glucose, insulin, IGF-1, hsCRP) were expressed as percentage change from baseline and averaged to obtain a single metabolic-stress score for each dietary condition. Similarly, clinical toxicity was quantified as the mean of all available toxicity measures reported in the respective trials.

Each clinical category was then matched to *in vitro* data obtained from tumor and healthy cells cultured under 3D static and 3D dynamic conditions and exposed to either conventional (High G) or fasting-mimicking therapy (No G): score 4/5 with tumor cell death (efficacy), SD/PD with tumor cell viability (non-response), CR/PR with decreased tumor metabolic activity (tumor vulnerability), composite toxicity with healthy cell death (toxicity), and composite metabolic stress score with metabolic stress in healthy cells.

For the comparative analysis, clinical and *in vitro* values were normalized to a 0–100 scale to enable direct visual comparison. Radar plots were generated using averaged values for each parameter within each condition (regular diet vs*.* fasting-mimicking diet). All analyses were performed in R or GraphPad Prism 10.3, and graphical outputs were produced using the *ggplot2* package.

### 4.10 Statistical analysis

Data were analyzed with GraphPad Prism 9.3 software. One-way ANOVA or two-way ANOVA were used for cells viability and cells metabolism measurements comparison between the different experimental groups. Level of significance was set at p < 0.05 (*p < 0.05, **p < 0.01, ***p < 0.001, ****p < 0.0001). The number of replicates and the specific statistical test for each experiment is reported in the figure legends. Data are shown as mean ± SD.

## Data Availability

The raw data supporting the conclusions of this article will be made available by the authors, without undue reservation.
